# Genetic Markers Associated with Field PRRSV-Induced Abortion Rates

**DOI:** 10.3390/v11080706

**Published:** 2019-08-01

**Authors:** Ramona N. Pena, Carlos Fernández, María Blasco-Felip, Lorenzo J. Fraile, Joan Estany

**Affiliations:** 1 Departament de Ciència Animal, Universitat de Lleida – AGROTECNIO Centre, 25198 Lleida, Spain; 2 INZAR, SL, 50840 Zaragoza, Spain; 3 Free-lance Veterinarian, 50001 Zaragoza, Spain

**Keywords:** PRRS, DNA markers, abortion rate, transplacental infection

## Abstract

In gilts and sows, the more severe clinical manifestation of porcine reproductive and respiratory syndrome virus (PRRSV) occurs in late gestation and can result in up to a 40% abortion incidence. Despite the known genetic component in resilience to PRRSV, there is scarce information regarding the abortive outcome of this disease. We tested the relationship between eight molecular markers (six from published studies and two identified in the present study in the *HDAC6* gene) and the probability of abortion during a PRRSV outbreak, using data from two commercial Landrace x Large White sow farms with an incidence of abortion of 35% and 17%. From the markers tested, *USP18*_-1533G>A did not segregate in these populations, and *CD163*_c.3534C>T and *HDAC6*_g.2360C>T did not affect the abortion rate. In contrast, the minor allele of two markers in SSC4 (WUR1000125 in *GBP1* and rs340943904 in *GBP5*), which lower viremia in growing pigs, and the major alleles of *CD163*_rs1107556229 and *HDAC6*_rs325981825 were associated with a lower probability of abortion during PRRSV outbreaks. The more striking result was for the *MX1* gene, where the odds ratio of aborting versus not aborting was nine times lower in the sows homozygous for a 275-bp insertion than in the other genotypes. Interactions between markers were not relevant. All together, we bring here the first evidence that mutations in the host genome can predispose or protect from complete reproductive failure in sows infected with PRRSV.

## 1. Introduction

Pigs of all ages are susceptible to porcine reproductive and respiratory syndrome virus (PRRSV). The clinical presentation of PRRS varies greatly between herds and can range from asymptomatic to devastating disease [1]. In gilts and sows, PRRSV infection can cause reproductive failure particularly in early and late gestation, when the virus has the ability to cross the placental wall and infect the embryos. Pregnancy in pigs lasts for 114–116 days. Embryos dead prior to implantation are generally resorbed, and sows return to estrus. Embryos are also probably resorbed when death occurs between 14 and 35 days of gestation, causing irregular return to estrus if all embryos die or small litters if some of the embryos survive [2]. The more severe clinical manifestation of PRRSV occurs in late gestation and is characterized by abortions (up to a 40% abortion rate in late-pregnancy sows); early farrowings; fetal death; and the birth of weak, congenitally infected piglets, resulting in elevated preweaning mortality [3]. The formation of mummies, which is characteristic of PRRSV late-term infection, occurs by exsiccation of a dead fetus. Besides reproductive failure, clinical signs in pregnant sows and gilts are often mild or absent.

The mechanisms of transplacental infection and why virus transmission is blocked midpregnancy are still unclear. Pigs develop an incomplete diffuse epitheliochorial placenta without invasion. Neither the invasion of fetal tissue into the maternal endometrium nor endometrial decidualization occurs, leaving a clear distinction between maternal and fetal tissues. In pigs, maternal and fetal blood is separated by six layers of tissue (maternal endothelium, endometrial connective tissue, uterine epithelium, trophoblast, fetal placental mesenchyme, and fetal endothelium), which form a firm barrier. This barrier prevents the crossing of antibodies and many microorganisms. However, vertical transmission for some microorganisms is feasible, and in the case of PRRSV, this transmission most likely relies on the migration of infected macrophages though this placental barrier. Although fetuses are susceptible to PRRSV at any stage of gestation upon direct intrafetal inoculation, transplacental PRRSV infection mainly occurs in late gestation [3]. The exact mechanism by which PRRSV transmits from the dam to her fetuses is not known, but it seems to reflect the varying numbers of CD169^+^ macrophages in the endometrium and placenta. Although CD163^+^ macrophages are present in these tissues during the whole pregnancy, CD169^+^ cells are rare in the placenta midgestation, which might explain the protection at this stage [2]. Once the fetal membranes are infected, the virus spreads and infects most fetal organs. In addition, recent studies have indicated that the virus readily infects neighbor fetuses, spreading the infection to the litter. A “Trojan-horse” model has been proposed for maternal macrophages to migrate from mother to fetus and then invade the full litter [2]. PRRSV-infected cells die of apoptosis, and this causes a gradual degradation of the maternal/placental junctions, leading to separation between the uterine epithelium and the trophoblast and eventually the loss of placental integrity. These serious histopathological lesions are not compatible with fetal life. To cause abortion or preterm birth, PRRSV probably induces severe lesions in the maternal–fetal interface of most, if not all, the fetuses. 

The severity of the reproductive failure in sows depends on the viral strain (pathogen virulence), the pregnancy stage of the female, the presence of neutralizing antibodies due to previous infection, and the general health status of the farm. In addition, several studies have evidenced that the final outcome also depends on the genetics of the female (host genetics). For instance, there is interbreed and intrabreed variation in humoral and cell-mediated immune responses, both of which are activated following exposure to the virus (reviewed in References [4] and [5]). The heritability of most of these responses is moderate or high [5]. All of this supports the hypothesis that animals exposed to the same pathogen in the same environmental conditions will develop different immune responses. Lewis et al. [6] showed that there was a genetic component to PRRSV resilience in sows, as indicated by the greater heritability of dead and mummified piglets observed during the epidemic phase of the disease compared to the healthy phase. 

A small number of genetic markers have been studied in relation to the respiratory [7,8,9] and reproductive [3,10] phases of PRRSV or the in vitro responses to PRRSV cell infection [11,12,13,14]. However, none have been assessed to date in relation to the incidence of abortion during a PRRSV outbreak. With this in mind, the objective of the present study was to use data from two resource populations to analyze the association between six previously reported PRRSV immune response markers and the likelihood of abortion in production sows (Table 1). In an effort to identify new potential markers associated with the incidence of abortion, we also described sequence variants in the *HDAC6* (histone deacetylase 6) gene given its involvement in controlling PRRSV replication [15]. This gene has been partially sequenced, and two polymorphisms have been studied in relation to the abortion rate. 

## 2. Materials and Methods

### 2.1. Pig Populations

Two populations of Landrace x Large White sows in commercial nursery farms in northeastern Spain were used for this analysis. Population 1 belonged to a 1500-PRRSV-naïve sow farm naturally infected with a field strain of the virus. The infection lasted for six weeks and resulted in an incidence of abortion close to 35% in two consecutive batches in the last third of the gestation period. Samples of jugular blood were collected from a subset of 180 sows (60 aborted and 120 not-aborted). No more data were available from this population. Population 2 was from a 1000-PRRSV-positive stable sow farm (following the classification proposed by Holtkamp in 2011 [17]) that underwent a sudden epidemic outbreak with an incidence of abortion close to 17% in the last third of the gestation period. Twelve sows aborted in the last third of the gestation period in this population, which were sampled along with 59 nonaborted females of the same batch of production to get a total of 71 samples. PRRSV genotype 1 (PRRSV-1), subtype 1, was involved in both clinical cases. The diagnosis was carried out by a laboratory specializing in pig diseases (http://www.gsplleida.net/ca) using standard operational procedures [18,19]. The homology between both viruses was probed to be quite low (89%), confirming that a different PRRSV strain was involved in each population [20].

### 2.2. DNA Isolation

Genomic DNA was isolated from blood by proteinase K/SDS lysis buffer, followed by phenol/chloroform extraction and isopropanol precipitation using standard techniques [21]. The concentration and purity of DNA was assessed by spectrometry using a Nanodrop-100 (Fisher Scientific, Madrid, Spain) and was standardized at 10 ng/µL per sample.

### 2.3. Primer Design and Genotyping Protocol

Six markers from published studies and two additional makers identified in the present study in the *HDAC6* gene were selected to be genotyped in the resource populations. Seven of these markers were single-nucleotide polymorphisms (SNPs). Six of them were genotyped using qPCR-HRM (high-resolution melting) technology (Supplementary Table S1) as follows. Sequences of the corresponding genes were exported from the latest version of the pig genome in Ensembl (*Sscrofa 11.1*) and were used to design PCR primers with the Primer3plus tool (https://primer3plus.com/cgi-bin/dev/primer3plus.cgi; [22]) using standard qPCR parameters. PCR reactions were set up in a volume of 7 µL containing 1× Luminaris HRM Master Mix (Fisher Scientific), 0.2 µM of each primer, and 10–15 ng/DNA. Reactions were run in a QuantStudio 3 thermocycler (Fisher Scientific) using the following program: initial denaturation at 95 °C for 10 min, 40 cycles of 95 °C for 10 s and 60 °C for 1 min, followed by a slow ramp from 60 °C to 95 °C at a rate of 0.015 °C/s. Genotypes were assigned by comparing PCR melting curves using the HRM software (Fisher Scientific). The *GBP1* SNP marker (rs80800372 or WUR1000125) was genotyped by allelic discrimination using allele-specific TaqMan probes labeled with FAM and VIC (Supplementary Table S1). For this, PCR reactions (7 µL) were set up with 1× TaqMan Universal PCR Master Mix (Fisher Scientific), a 1× Primer and Probe set, and 10–15 ng/DNA. Reactions were run in a QuantStudio 3 thermocycler using the following program: initial fluorescence detection at 60 °C for 1 min, denaturation at 95 °C for 10 min, 40 cycles of 95 °C for 10 s and 60 °C for 1 min, and final fluorescence detection at 60 °C for 1 min. Genotypes were assigned by comparing the accumulation of FAM and VIC fluorescence in each channel.

The eighth marker was a 275-bp insertion in the promoter of the *MX1* gene, which was genotyped with an end-point PCR and electrophoresis in an agarose gel. A PCR reaction was set up in a volume of 15 µL containing 1× buffer, 0.2 mM dNTPs, 2 mM MgCl_2_, 0.4 µM of each primer, and 0.4 U of Taq polymerase (Bioline, London, UK). The primers are summarized in Supplementary Table S1. 

### 2.4. HDAC6 Characterization and Genotyping

The *HDAC6* gene sequence was exported from Ensembl as described above and was used for primer design with Primer3Plus using standard PCR parameters and with the desired PCR product size set in the 600–1200-bp range. Primer sets were designed to amplify five fragments of the gene that included most of the exons (Supplementary Table S2). This gene (~25 Kb) has 31 exons, most of them of a small size, that are spliced into five transcript variants leading to the production of two proteins differing in the last 50 amino acids. The PCR fragments were selected to include as much of the coding region as possible. 

To describe new mutations in this gene, the five fragments were amplified and sequenced in 16 sows, 8 aborting and 8 nonaborting, from Population 1. PCR reactions included 1× buffer, 160 mM dNTPs, 1.5 mM MgCl_2_, 0.4 µM of each primer, 50 ng of genomic DNA, and 1 U of Taq polymerase (Bioline) in a final volume of 25 µL. The following thermal program was run in a Veriti thermocycler (Applied Biosystems, Foster City, CA, USA): initial denaturation at 95 °C for 5 min, 35 cycles of 95 × 20 s, 58 °C × 40 s, 72 °C × 1 min, and a final extension at 72 °C for 5 min. PCR reactions were cleaned with NZYGelpure columns (NZYtech, Lisbon, Portugal) prior to Sanger sequencing. The software Chromas Pro (Technelysium Pty Ltd, South Brisbane, Australia) was used to compare the sequences and identify new mutations. SIFT and PPolyphem-2 were used to predict the functional consequences of new mutations in protein functionality. Haplotype frequencies and linkages between markers in the same chromosome were estimated with Haploview [23].

### 2.5. Statistical Analysis

The “abortion during PRRSV outbreak” outcome (aborted vs not-aborted) was analyzed using a binary logistic regression model on each genotype and population. The probability of aborting during a PRRSV outbreak was calculated as follows:

(1) $$P\left(y=j\right)=\frac{e^{\alpha +b\prime x_{i}}}{1+e^{\alpha +b\prime x_{i}}}$$

where *P*(*y* = *j*) is the probability of occurrence of the abortion condition *j* in a specified situation *i*; α is a constant; and *b’* and *x_i_* are, respectively, the vector of coefficients in the logistic equation and the vector of independent variables in situation *i*. The genotypes for each individual investigated gene and the farm were included as independent variables. To test for potential associations and interactions between genotypes, the same model considering two genotypes at a time (all pairs of genotypes were tested) and their interactions was also used. The effects of each genotype and the corresponding odds ratios were tested using a χ^2^ approximation to the asymptotic distribution of the likelihood ratio test. Analyses were performed with JMP Pro14 (SAS Institute Inc., Cary, NC, USA) software.

## 3. Results

### 3.1. Characterization of Sequence Variants in Pig HDAC6

The five fragments of the *HDAC6* gene were successfully sequenced in two groups of sows, which either aborted or not during a PRRSV outbreak. A sequence comparison identified 10 SNPs, 6 of them in the coding sequence (Table 2). Three of these polymorphisms changed the sequence of the encoded protein at the amino acid positions 12, 360, and 503. Two protein intolerance prediction tools catalogued these mutations as potentially damaging for the functionality of the protein (Table 3), particularly the last two, as they were in the catalytic domain of the protein and in very conserved residues when compared to other species. Genotyping protocols were set up for the three mutations using an HRM-qPCR approximation. However, the Pro503His could not be genotyped with enough quality and was discarded from future analysis.

### 3.2. Allelic and Genotypic Frequencies of the Markers

The eight polymorphisms tested (Table 1) segregated in the two populations used for this study except for the *USP18* -1533G>A marker, which was fixed at the G allele. This mutation, located in the promoter of the *USP18* gene (ubiquitin specific protease 18), enhances the expression of this gene, probably by altering a binding site of the FOX transcription factor family [16]. In vitro *USP18* overexpression can stop PRRSV infection in MARC-145 cells [14]. The fixed G allele agreed with data from Li et al. [23], who observed segregation of the A allele in three Asian pig breeds but not in Landrace, Duroc, or Yorkshire breeds. 

Genotypic information from the two farms was pooled to calculate allelic frequencies. Overall, the minor allele frequencies were low or moderate, ranging from 0.19 for the *GBP1* mutation to 0.38 for the g.2360C>T SNP in *HDAC6*. The distribution of genotypes is indicated in Table 4. The linkage disequilibrium between markers in the same chromosome was high for *GBP1*–*GBP5* (D’ = 0.89) and moderate and low for the two polymorphisms at *HDAC6* (D’ = 0.62) and *CD163* (D’ = 0.32).

### 3.3. Association Study between Markers and Abortion Rate

**Figure 1:**
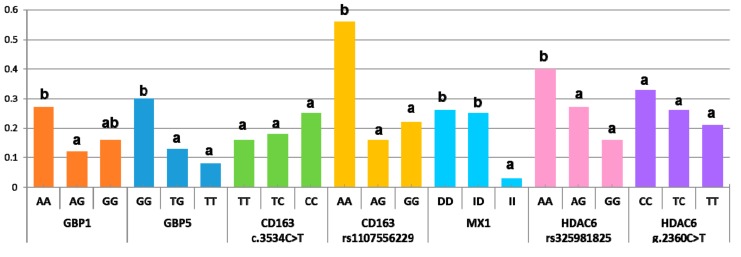
Abortion probability during a PRRSV outbreak by marker genotype (data pooled from the two studied populations). Within each marker, the genotypes with different superscripts indicate differences in the abortion ratio (*p* < 0.05).

During the PRRSV outbreak, the probability of abortion was significantly different in homozygous sows for the minor alleles of *GBP1*, *GBP5*, *CD163* (rs1107556229), *MX1*, and *HDAC6* (rs325981825) (Figure 1 and Supplementary Table S3; *p* < 0.05). For the *GBP1* marker, the odds ratio for abortion versus not was 2.69 times higher in AA sows than in AG sows (Table 5). In the *GBP5* marker, this odds ratio was 2.76 to 4.49 times higher in the GG genotype than in the TT and TG genotypes (Table 5; *p* < 0.05). Similarly, for the HDAC6_rs325981825 genotype, the GG sows had an odds ratio 2–2.5 times lower than in AA and AG sows (*p* < 0.05). A more extreme situation happened in the *MX1* markers, where the homozygous animals with the minor allele showed an odds ratio for abortion versus not of 9 times less than the other two genotypes (*p* < 0.05). For the CD163_rs1107556229 marker, the minor allele (GG genotype) was associated with an odds ratio 1.96 to 3.6 times higher than in the AG and AA genotypes. Abortion rates did not differ between the CD163_c.3534C>T and HDAC6_g.2360C>T genotypes. On the whole, minor alleles for *GBP1*, *GBP5*, and *MX1* markers and major alleles for CD163_rs1107556229 and HDAC6_rs325981825 had a protective role against total reproductive failure in sows exposed to a sudden outbreak of this disease. Interestingly, the effect of each genotype did not substantially change when they were analyzed in groups of two, with the exception of *GBP1*, whose impact on the abortion rate vanished when *GBP5* was also included in the model. This reinforces the role of *GBP5* in resilience to PRRSV compared to *GBP1*, whose effect should be explained by the strength of its linkage disequilibrium with *GBP5*. No significant interactions were observed besides that between GBP1_WUR10000125 and HDAC6_rs325981825 (*p* < 0.03), which, however, was not detected when *GBP1* was replaced with *GBP5*. Therefore, interactions between markers did not play a relevant role in predicting the likelihood of abortion during a PRRSV outbreak.

## 4. Discussion

In previous studies led by the PRRS Host Genomic Consortium (PHGC), large sample sizes of the pregnant gilt model enabled an exceptional opportunity to identify new genomic regions associated with reproductive PRRS outcomes [3,8,24,25,26]. Twenty-one candidate genomic regions across 10 chromosomes were found to be significantly associated with fetal viability, fetal death, and viral load in the fetal thymus. Together, these regions accounted for a biologically relevant portion of the overall genetic variation. Seven of these overlapped with previously reported quantitative trait loci (QTLs) for pig health and reproduction [27]. However, it was not feasible to describe any marker associated with abortion because the abortion rate in this experimental model was unexpectedly low. Recently, Scanlan and coworkers [28] estimated the heritability of abortion rates, which was low (0.07) in healthy farms but rose to 0.17 during PRRSV outbreaks. In this context, our research work provides the first scientific evidence that the genetic background of the sow could be linked with the probability of abortion after a PRRSV infection.

The first two molecular markers tested were rs80800372 (i.e., WUR10000125) in *GBP1* and rs340943904 in *GBP5* (Table 1). WUR10000125 was the first molecular marker associated with PRRSV resistance and productivity. A genome-wide association study identified a genomic region in SSC4, represented by WUR10000125, which explained 15.7% of the genetic variance in viral load and 11.2% of weight gain in weaned piglets directly challenged with a very pathogenic American (PRRSV-2) strain [7]. The estimated effects for this region were favorably and nearly perfectly correlated: That is, pigs with a low virus load exhibited greater weight gain. The WUR10000125 mutation is a single-nucleotide polymorphism (SNP) located in the 3’UTR region of the *GBP1* gene, a response gene to type II interferons. The G allele modifies a close polyadenylation signal in the *GBP1* gene, reversing the proportion of two alternative transcripts [29]. The clinical and productive outcomes of this marker have been validated in experimental [7,30] or natural challenges [8,18] with PRRSV-1 [18] and -2 in growing animals [7,10,30,31]. However, the causality of this region of chromosome 4 is currently attributed to a nearby gene, *GBP5*, where an intronic SNP (rs340943904) (also included in our study) generates a new splicing acceptor site, changing the proportion of two alternative transcripts [9]. This gene encodes for a protein involved in the inflammasome assembly during innate immune responses [7,9].

The effect of the WUR10000125 genotype on reproductive traits is controversial. An initial study found that sows carrying the favorable G allele had more piglets born alive and weaned than homozygous AA sows in uninfected farms [12]. However, this result was not replicated in a subsequent experimental infection using a PRRSV-2 strain. The WUR10000125 genotype in both gilts and fetuses was associated neither with fetal death/viral load [32] nor with reproductive performance during a PRRSV outbreak in a commercial multiplier sow herd [11]. However, we describe here that, as for the respiratory form of the disease, the G allele was outstanding as a protective factor against abortion (Figure 1). The difference in abortion rate probability was more evident in the closely linked *GBP5* marker. In this case, the G allele showed an additive pattern, as described previously for WUR10000125 in the respiratory form of the disease [7,18]. Thus, the genotype GG was more susceptible to abortion (probability of abortion 30%) than GT and TT sows (the less represented genotype), where the probability of abortion was 13% and 3%, respectively. Our results complement previous studies on the effect of the SSC4 region on reproductive traits, where abortion rates were not included. It must be highlighted that, by studying the abortion rate and not the reproductive performance of nonaborted sows, we probably discriminated the sow population most susceptible to the virus.

Our study also included two markers in the *CD163* gene (Table 1), which encodes the membrane receptor used by the virus to enter macrophages and initiate infection [1]. Several natural mutations have been described in this gene, some of which have been related to a better response capacity of pigs to the virus. For example, the CC genotype of the CD163_c.3534C>T polymorphism has been associated with lower levels of IgG [33] and viremia and enhanced weight gain [11] after a PRRSV challenge. The second polymorphism selected in this gene, c.2494G>A (rs1107556229), is located at the end of exon 10 and is a synonymous variant catalogued as a splice region variant by the variant effect predictor (VEP) tool. Our results clearly show that the different variants of CD163_rs1107556229 were linked with the probability of abortion in infected sows (Table 5). Scavenger receptor CD163 is a key entry mediator for PRRSV, and complete [34] or extracellular domain [35] deletion by genetic editing of this receptor makes pigs fully resistant to PRRSV infection. Thus, mutations in *CD163* could imply a significant change in the susceptibility of the pigs to PRRSV infection. Our results totally agree with this information in the sense that sows with the rs1107556229 variant, naturally present in nature, had a different clinical outcome when abortion was the end-point measure (Table 5 and Figure 1). 

The next gene marker tested was a 275-bp insertion in the proximal promoter of the *MX1* gene (MX Dynamin Like GTPase 1), which enhances the transcriptional activity of this gene, mediating the humoral response to infection with PRRSV [13]. As with *GBP1* and *GBP5*, *MX1* encodes a guanosine triphosphate (GTP)-metabolizing protein, whose expression is induced by interferons. MX1 has strong antiviral activity against a wide range of RNA viruses and some DNA viruses through binding and inactivation of their ribonucleocapsid. The antiviral effect of MX1 against the influenza virus has been well documented. Mice carrying the defective *MX1* gene show greater susceptibility to influenza A infection compared to mice carrying the functional protein [36]. MX1 also conferred protection against the influenza A virus, classical swine fever virus, and vesicular stomatitis virus in studies performed in vitro [37]. The mutation studied here was associated with higher *MX1* expression in vitro [13]. In our data, this mutation showed the strongest association with a low abortion rate (Table 5). The effect was completely recessive, where sows with two copies of the insertion had an abortion probability of 3% during the outbreak, while in the other sows the probability was ~25% (Figure 1). As with the favorable alleles of *GBP1* and *GBP5*, the MAF of this allele was low (<0.20, Table 4), which raises the question of whether there was an indirect selection of these alleles representing three genes with very similar functionalities. Selection in nucleus farms is performed in clean environments with a lower pathogen load than in standard field conditions. This finding supports also studying the possible effect of these markers in healthy production farms and in other production traits.

The last gene studied was *HDAC6*. This histone deacetylase gene was selected for its role in macrophage differentiation. Initially, histone deacetylases were characterized as enzymes that removed acetyl groups from histones, establishing a silent chromatin structure. However, HDACs have recently been shown to act over a wider spectrum of substrate proteins, involved in a range of cellular processes that extend beyond epigenetic labels. Histone deacetylases are critical regulators in macrophage differentiation and in maintaining M1 or M2 macrophage functions in Th1 and Th2 T-cell responses, respectively. The balance between the contrasting cytokine profiles of M1 and M2 macrophages regulates many immune checkpoint modulators [38,39]. *HDAC6* overexpression in cells and in transgenic mice enhances resistance to viral infection with the human acquired immunodeficiency virus (HIV-1), influenza A virus, and vesicular stomatitis virus [40]. Recently, *HDAC6* overexpression in transgenic pigs reduced the viral load of PRRSV-challenged animals and had extended survival time and fewer clinical signs than wild-type controls [15]. We identified three missense mutations in the coding region of this gene (Table 2) with potential damaging effects on the function of the protein, two of them directly affecting the deacetylase domain of the protein (Table 3). The two markers analyzed had intermediate allelic frequencies, with MAF close to 0.40 (Table 4). The odds ratios of aborting versus not aborting during an outbreak were 2.3 to 4 times greater in sows of the rs325981825 GG genotype than in the other genotypes (Table 5). The probability of abortion followed an additive pattern, similar to GBP5_rs340943904, where the presence of the A allele raised the probability of abortion from 16% in GG sows to 27% and 40% in AG and AA animals, respectively (Figure 1). Given the intermediate frequencies, this marker responded better to selection than the other three favorable alleles did, which were present at a much lower frequency. 

## 5. Conclusions

In conclusion, we bring here the first evidence that mutations in the host genome can predispose or protect from complete reproductive failure in sows infected with a field strain of PRRSV. Although transcriptomic and genomic scans have studied the genetic component of spontaneous abortions in pigs [41] and cattle [42], abortion as an outcome of an infectious disease has not been explored from a genetic point of view in pigs. The low or intermediate allelic frequency of some protective alleles and potential interactions between some of them will need to be tackled in future studies.

## Figures and Tables

**Table 1:** Information about the candidate genes and polymorphisms examined in the present study.

Marker	Gene Acronym	Gene Function	Polymorphism	Gene Location	Chromosomal Location ^1^	Reference
rs80800372	*GBP1*	Interferon-induced guanylate binding protein with known antiviral functions	A>G	3’UTR	SSC4	[7]
rs340943904	*GBP5*	Inflammasome assembly, innate immunity	G>T	Intron 5	SSC4	[9]
c.3534C>T	*CD163*	Macrophage-specific scavenger receptor, mediates PRRSV entry into macrophages	C>T	3’UTR	SSC5	[11]
rs1107556229			G>A	Exon 10	SSC5	[11]
-547ins+275	*MX1*	Interferon-induced GTP metabolizing enzyme, antiviral properties	Indel 275 bp	Promoter	SSC13	[12]
-1533G>A	*USP18*	Ubiquitin-specific proteases, Downregulation of interferon responses	G>A	Promoter	SSC5	[16]
rs325981825	*HDAC6*	Epigenetic labeling of histones by acetylation/deacetylation	G>A	Exon 3	SSCX	This study
g.2360C>T			C>T	Exon 15	SSCX	This study

^1^ SSC: *Sus scrofa* chromosome; PRRSV: porcine reproductive and respiratory syndrome virus.

**Table 2:** Polymorphisms found in the sequenced fragments of the *HDAC6* gene.

*HDAC6* Fragment	Polymorphism	Position from ATG *	Location	Change Type
Fragment 1	C/T	−1538	Exon 1	5′UTR
Fragment 2	G/A	+35	Exon 3	Missense (Arg12Lys)
	C/G	+63	Exon 3	Synonym (His21)
Fragment 3	G/A	+2180	Intron 13	-
	G/A	+2222	Exon 14	Synonym (Gln337)
	G/A	+2340	Intron 14	-
	C/T	+2360	Exon 15	Missense (Pro360Leu)
Fragment 4	C/A	+3785	Exon 19	Missense (Pro503His)
Fragment 5	G/T	+9813	Exon 25	Synonym (Gln799)
	C/A	+10450	Intron 26	-

* The position of the polymorphisms was calculated over the genomic DNA sequence taking the position of the start codon as a reference.

**Table 3:** Functional predictions in the missense mutations of the *HDAC6* gene.

Polymorphism	Protein Domain (EMBL-EBI)	SIFT Prediction (Score)	Polyphen-2 Prediction (Score)
Arg12Lys		Not tolerant (0.00) *	Unknown, not enough reference sequences
Pro360Leu	Hist_deacetyl (PF00850)	Not tolerant (0.02)	Probably damaging (1.000)
Pro503His	Hist_deacetyl (PF00850)	Not tolerant (0.00)	Probably damaging (1.000)

* Detected with low confidence, as there were few proteins in the database that included this residue.

**Table 4:** Allelic and genotypic frequencies of the eight markers studied in the sows used in this study.

Marker	Gene	MAF (Allele)	AA	AB	BB *
rs80800372	*GBP1*	0.19 (G)	15	64	171
rs340943904	*GBP5*	0.25 (T)	22	82	144
c.3534C>T	*CD163*	0.33 (C)	41	70	119
rs1107556229	*CD163*	0.29 (A)	32	70	127
−547ins+275	*MX1*	0.25 (insertion)	18	50	170
rs325981825	*HDAC6*	0.37 (A)	35	103	90
g.2360C>T	*HDAC6*	0.38 (T)	32	104	32

* A and B refer to minor and alternative alleles, respectively.

**Table 5:** Odds ratios of abortion versus no abortion probabilities during a PRRSV outbreak by marker genotype. Only markers showing different abortion rates by genotype are shown.

Marker	Gene	Contrast	Odds Ratio	*p*
rs80800372	*GBP1*	AA/AG	2.69	0.008
rs340943904	*GBP5*	GG/TG	2.76	0.003
		GG/TT	4.49	0.02
rs1107556229	*CD163*	AA/AG	2.58	<0.0001
		AA/GG	1.96	0.0004
−547ins+275	*MX1*	DD/II	9.35	0.03
		ID/II	8.63	0.04
rs325981825	*HDAC6*	AA/GG	4.08	0.002
		AG/GG	2.34	0.02

## Supplementary Materials

The following are available online at https://www.mdpi.com/1999-4915/11/8/706/s1, Table S1: Primers and probes used in the genotyping experiment; Table S2: Primers used to sequence the *HDAC6* promoter and coding regions; Table S3: Comparison of the probability of abortion during a PRRSV outbreak by marker genotype in the sow population studied.
