# Supplementary material for: Genetic Markers Associated with Field PRRSV-Induced Abortion Rates
Source: Viruses. 2019 Aug 1;11(8):706. doi: 10.3390/v11080706 (PMC6723062; doi:10.3390/v11080706)
Supplement: Supplementary file 1 [file viruses-11-00706-s001.pdf]

Supplementary tables

Supplementary Table S1. Primers and probes used in the genotyping experiment.

Marker	Gene	Primer sequence (5′→3′)	Genotyping protocol
rs80800372	*GBP1*	Fw: AGACCTAGAATCTCCACAGAATTTCCA<br>Rv: GGAAAGGACAGTTCGCTTCTCTAG<br>Probe-A: VIC-CTGGGTGATAAATAAAT-NFQ[1]<br>Probe-G: FAM-TGGGTGATGAATAAAT-NFQ[1]	Allelic discrimination
rs340943904	*GBP5*	Fw: AGGATGGCTGCTAACACTTG<br>Rv: GGAGGGAGAAGGATGGGTACT	HRM-qPCR
c.3534C>T	*CD163*	Fw: CTCCTGGTATTCCAAAGACTGC<br>Rv: GAAAATCAAGAACAAGAATCAGCA	HRM-qPCR
rs1107556229	*CD163*	Fw: ATTGCAGGCATAAGGAGGATG<br>Rv: GAGGTGATGTGCAGAACTTACC	HRM-qPCR
-547ins+275	*MX1*	Fw: CCAGATTGCCAAATTCCCTA<br>Rv: CAGGCTAGGGGTTGACTCAG	End-point PCR
-1533G>A	*USP16*	Fw: ATGCAGGAAAGAAGGGAAGG<br>Rv: GGGACATGAACACAAACACG	HRM-qPCR
rs325981825	*HDAC6*	Fw: GAAGTCTCCCCAGCCATGAC<br>Rv: TGGGGGTTGTGCCTACTTC	HRM-qPCR
g.2360C>T	*HDAC6*	Fw: TTCTCAGTCACCTCCATCCC<br>Rv: GGTTGTGCCTACTTCTTCGC	HRM-qPCR

[1] NFQ – non-fluorogenic quencher

Supplementary Table S2. Primers used to sequence the *HDAC6* exons.

Primer name	Target region	Primer sequence (5′→3′)	PCR Length
**HDAC6-Fg1**	Exon 1	Fw: CATGGGGTGGGAGAGAGTT<br>Rv: GCGGTTTCTTTGTCCCTGT	727bp
**HDAC6-Fg2**	Exons 3-5	Fw: GATGGAGGCTAGCGTAGGG<br>Rv: TGAGTATGGCCTGGAGAAGC	695bp
**HDAC6-Fg3**	Exons 12-15	Fw: TGGGGGAACTAAGGAGAGAA<br>Rv: CCCACTTAACTCAGGGATCG	862bp
**HDAC6-Fg4**	Exons 16-20	Fw: CCCTCACATTCCCCCTCCTA<br>Rv: CCTCTCCTGGAGCCTAAGGT	1287bp
**HDAC6-Fg5**	Exon 25-27	Fw: TCCAGTCTCCCCAGTTGTCT<br>CACAAACGGGCCAAAGAAGG	997bp

Supplementary Table S3. Comparison of the probability of abortion during a PRRSV outbreak by marker genotype in the sow population studied.

Marker	Gene	p-value	AA	AB	BB
rs80800372	*GBP1*	**0.01**	0.16	0.12	0.27
rs340943904	*GBP5*	**0.002**	0.08	0.13	0.30
c.3534C>T	*CD163*	0.35	0.16	0.18	0.25
rs1107556229	*CD163*	**0.0001**	0.56	0.16	0.22
-547ins+275	*MX1*	**0.01**	0.03	0.25	0.26
rs325981825	*HDAC6*	**0.003**	0.40	0.27	0.16
g.2360C>T	*HDAC6*	0.43	0.33	0.26	0.21

**A and B refer to minor and alternative allele, respectively*
